# Activation of the μ‐opioid receptor by alicyclic fentanyls: Changes from high potency full agonists to low potency partial agonists with increasing alicyclic substructure

**DOI:** 10.1002/dta.2906

**Published:** 2020-08-14

**Authors:** Anna Åstrand, Svante Vikingsson, Ingrid Jakobsen, Niclas Björn, Robert Kronstrand, Henrik Gréen

**Affiliations:** ^1^ Division of Drug Research, Department of Biomedical and Clinical Sciences, Faculty of Medicine and Health Sciences Linköping University Linköping Sweden; ^2^ Department of Forensic Genetics and Forensic Toxicology National Board of Forensic Medicine Linköping Sweden; ^3^ Department of Laboratory Medicine Örebro University Hospital Örebro Sweden

**Keywords:** cyclopropylfentanyl, fentanyl analogs, NPS, potency, μ‐opioid receptor agonist

## Abstract

Fentanyl analogs represent an important group of new psychoactive substances and knowing their efficacy and potency might assist in interpreting observed concentrations. The potency of fentanyl analogs can be estimated from in vitro studies and can be used to establish structure–activity relationships. In this study, recombinant CHO‐K1 cells (AequoScreen) expressing the human μ‐opioid receptor were used to establish dose–response curves via luminescent analysis for cyclopropyl‐, cyclobutyl‐, cyclopentyl‐, cyclohexyl‐, and 2,2,3,3‐tetramethylcyclopropylfentanyl (TMCPF), on three separate occasions, using eight different concentrations in an eight‐fold serial dilution in triplicates starting at ~60 μM. Fentanyl was used as a full agonist reference while morphine and buprenorphine were included for comparison. Cyclopropylfentanyl (EC_50_ = 4.3 nM), cyclobutylfentanyl (EC50 = 6.2 nM), and cyclopentylfentanyl (EC_50_ = 13 nM) were full agonists slightly less potent than fentanyl (EC_50_ = 1.7 nM). Cyclohexylfentanyl (EC_50_ = 3.1 μM, efficacy 48%) and TMCPF (EC_50_ = 1.5 μM, efficacy 65%) were partial agonists less potent than morphine (EC_50_ = 430 nM). Based on the results, cyclopropyl‐, cyclobutyl‐, and cyclopentylfentanyl would be expected to induce intoxication or cause fatal poisonings at similar concentrations to fentanyl, while the toxic or fatal concentrations of cyclohexylfentanyl and TMCPF would be expected to be much higher.

## Graphical abstract

Cyclopropyl‐, cyclobutyl‐ and cyclopentyl‐fentanyl are full agonists with potency similar to fentanyl at the µ‐opioid receptor while Cyclohexyl‐ and 2,2,3,3‐tetramethyl‐cyclopropyl‐fentanyl (TMCPF) are partial agonists with potency lower than buprenorphine using an AequoScreen assay.

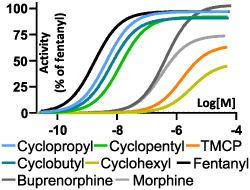

## 1 INTRODUCTION

Fentanyl analogs, belonging to the opioids, represent an important group of new psychoactive substances (NPS), especially when looking at drug overdoses and the number of deaths.^1^ Opioids exert their effects by activating the three major types of opioid receptors (μ, δ, and κ).^2^ However, the primary receptor involved in opioid addiction and the often lethal respiratory depression caused by opioid overdose is the μ‐opioid receptor.^2^, ^3^

In cases of suspected intoxication, knowing the efficacy and potency of the NPS might assist toxicologists and medical examiners in their interpretation. Similarly, predictions of potency based on the structure could be of value in scheduling decisions on new uncharacterized NPS. Potency can be studied in several ways, including functional studies in laboratory animals^4^ or receptor‐based studies in vitro. The latter can be divided into receptor binding assays and receptor activity studies. Receptor binding studies measure the affinity of a ligand to the opioid receptor^5^, ^6^ and are suitable for high throughput. However, binding studies cannot differentiate between full and partial agonists, and even an antagonist such as naloxone shows binding affinity.^6^ Receptor activation assays instead measure proximal or downstream activation of the receptor, allowing the potency and efficacy values for a certain agonist to be determined, making them more informative than binding assays. Receptor activation can be measured via different assays based on, for example, aequorin,^7^ GTPγS binding,^8^, ^9^ cAMP^10^, ^11^ and β‐arrestin recruitment.^12^ The interpretation of the data is complicated by the fact that potency is substantially affected by the assay conditions such as the reagent concentration, which reporting system was used, the cell type, and if a human or murine receptor was used, making it difficult to compare the results from different assays.

**FIGURE 1:**
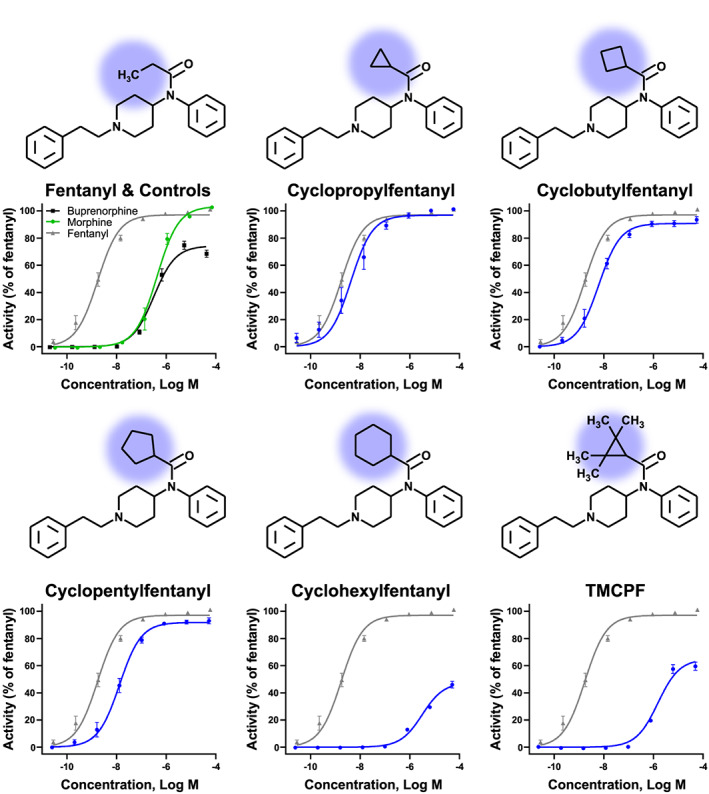
Chemical structures and dose–response curves of the alicyclic fentanyls (blue circles) in comparison with fentanyl (gray triangles) and known agonists buprenorphine (black squares) and morphine (green circles). Each point is the average of nine replicates except fentanyl (n = 21). Error bars represent standard error of the mean (SEM). TMCPF, 2,2,3,3‐tetramethylcyclopropylfentanyl [Colour figure can be viewed at wileyonlinelibrary.com]

Alicyclic fentanyls share their overall chemical structure with fentanyl, except for the substitution of the propionamide with amides with increasingly large cyclic structures containing either a cyclopropyl‐, cyclobutyl‐, cyclopentyl‐, cyclohexyl‐, or 2,2,3,3‐tetramethylcyclopropyl ring (see Figure 1). In 2017, cyclopropylfentanyl was reported in 59 deaths in Sweden.^13^ Cyclopentylfentanyl^14^ and 2,2,3,3‐tetramethylcyclopropylfentanyl (TMCPF) have also been encountered as NPS in Sweden.^15^ Similarly, cyclopropylfentanyl findings have been reported in the USA^16^, ^17^, ^18^ and Switzerland.^19^ Information about the pharmacokinetics of alicyclic fentanyls is limited but we know that they are metabolized differently in biological systems.^15^

The structure–activity relationships for fentanyl analogs have been investigated and reviewed.^12^, ^20^, ^21^, ^22^, ^23^ The binding affinity of cyclopropylfentanyl has been reported as 0.088, 1.2 0.77, and 2.4 nM in different assays.^5^, ^23^, ^24^ In addition, Hassanien et al^23^ reported the binding affinities for cyclobutyl‐ and cyclopentylfentanyl as 6.2 and 13 nM, respectively. Hassanien et al^23^ also reported EC_50_ values from their GTPγS binding assay as 55, 160, and 600 nM for cyclopropyl‐, cyclobutyl‐, and cyclopentylfentanyl, respectively. The corresponding efficacies were 75%, 61%, and 41% (compared with 10 μM [D‐Ala2, N‐MePhe4, Gly‐ol]‐enkephalin, DAMGO). This can be compared with an EC_50_ for cyclopropylfentanyl of 11 nM reported by the Drug Enforcement Administration,^21^, ^24^ also from a GTPγS binding assay. Finally, to study biased agonism, Vasudevan et al^12^ used two similar assays based on the recruitment of a G‐protein (mini‐G_i_) or β‐arrestin to study cyclopropyl‐ and cyclopentylfentanyl, and TMCPF. For the mini‐G_i_ assay they reported EC_50_ values of 42 and 190 nM, with efficacies of 280% and 159% (compared with hydromorphone), for cyclopropyl‐ and cyclopentylfentanyl, respectively. Similarly, for the β‐arrestin assay they reported EC_50_ values of 15 and 180 nM, with efficacies of 158% and 127% (compared with hydromorphone), for cyclopropyl‐ and cyclopentylfentanyl, respectively. For TMCPF, no signal was observed in either assay.

Previous literature indicates that both the potency and the efficacy differ between the different alicyclic fentanyl analogs but it is difficult to ascertain how the ring size impact these parameters. Therefore, the aim of this study was to determine the μ‐opioid receptor activity induced by a complete series of alicyclic fentanyls and to investigate the structure–activity relationships regarding potency and efficacy.

## 2 MATERIALS AND METHODS

### 2.1 | Drugs and chemicals

Reference standards of cyclopropyl‐, cyclobutyl‐, cyclopentyl‐, cyclohexyl‐, and TMCPF were purchased from Cayman Chemicals (Ann Arbor, MI, USA). The cell culture medium used was DMEM/Ham’s F12 with 15 mM HEPES, L‐glutamine, and without phenol red, from Thermo Fisher (Gothenburg, Sweden). Digitonin, ATP, trypsin, and protease‐free BSA were from Sigma‐Aldrich (Darmstadt, Germany). Fetal bovine serum (FBS) was from Life Technologies, Thermo Fisher (Gothenburg, Sweden). Coelenterazine was from Nanolight Tech (Pinetop, AZ, USA). Stock solutions of 500 μM coelenterazine were prepared in methanol (and protected from light), 50 mM digitonin in DMSO, and 10 mM ATP in Milli‐Q water and stored at −20°C

### **2.2 |** Cell lines and cultivation

The receptor activation assay was carried out on AequoScreen recombinant CHO‐K1 cell lines purchased from Perkin Elmer (Groningen, the Netherlands) expressing the human μ‐opioid receptor (ES‐542‐AV) and subunit Gα16 coupling receptor activation to an increase in intracellular Ca^2+^ concentration.^25^ The cells also expressed apoaeqourin which, when combined with externally added coelenterazine, forms the photoprotein aequorin. When aequorin is exposed to Ca^2+^, coelenterazine is oxidized with the emission of light.^26^ The flash luminescence can easily be read by a plate reader. When combined, the μ‐opioid receptor coupled to Gα16 and aequorin provide a convenient model system for measuring receptor activation. The cell lines were cultured at 37°C in a humidified air atmosphere containing 5% CO_2_, in Ham’s F12 medium supplemented with 10% FBS and passaged every 3–4 days. The cells were not cultured beyond 30 passages.

### **2.3 |** Dose–response assay

Prior to the dose–response assays, the cells were cultured to a confluency of 70–90% and then trypsinized, centrifuged (150 × g for 5 min at room temperature), and resuspended in pre‐warmed assay medium (DMEM/Ham’s F12 without phenol red supplemented with 15 mM HEPES, L‐glutamine, and 0.1% protease‐free BSA) at a concentration of 3 × 10^5^ cells/mL. Coelenterazine was added to the cells to a concentration of 2.5 μM. The cells were gently incubated on a rotating wheel at room temperature for 3 hours while protected from light. The alicyclic fentanyls were prepared in 96‐well plates (OptiPlate‐96, white opaque microplates from PerkinElmer), in triplicate, at final concentrations of 20,000, 4,000, 800, 160, 32, 6.4, 1.28, 0.256, 0.0512, 0.01024 ng/mL in each well (concentrations after adding the cells). Fentanyl, morphine, and buprenorphine were analyzed in the same concentration range to serve as μ‐opioid receptor full‐ and partial agonist references. As positive controls digitonin (67 μM) and ATP (7 μM) were used. Wells containing cells and medium without drug were used as negative controls. The receptor activation at each drug concentration was determined by dispensing 50 μL of cells into each well (15,000 cells/well) using a TECAN Spark 10 M (Tecan, Switzerland) for the subsequent luminescence reading. The reading protocol was set to 200 luminosity readings, and the cells were added to each well at reading cycle 10 (baseline). Luminescence reading was carried out for ~25 s before moving on to the next well.

The experiments for each substance were repeated on three different days comprising 6 days of analysis. Fentanyl was included as a reference every day and the data set for fentanyl contains seven experiments, including two from the same day.

### **2.4 |** Data analysis

Luminescence data from each well were summarized over the total reading time and blank measurements were subtracted. The response signals were normalized to the digitonin signal for each plate and then normalized to the plateau signal of fentanyl (average of top two concentrations analyzed in the same experiment), denoted as 100% activity.

The EC_50_ values and efficacy with 95% confidence intervals (profile likelihood) and curve fittings (non‐linear fit, three parameters, bottom constrained to 0%) were calculated using all data points (n = 72, fentanyl n = 168) using GraphPad Prism version 8.3.0 for Windows (GraphPad Software, La Jolla, CA, USA). The efficacy (derived as the top value from the linear regression) of all fentanyl analogs was compared with that of the full agonist fentanyl (5 comparisons, n = 3; fentanyl n = 7) using a one‐way ANOVA with Dunnett correction for multiple tests. Differences in potency, as Log (EC_50_) values from the regression, were compared between fentanyl and all fentanyl analogs (15 comparisons, n = 3; fentanyl n = 7) using a one‐way ANOVA with Dunnett correction for multiple tests.

## **3 |** RESULTS

Full dose–response curves were obtained for cyclopropyl‐, cyclobutyl‐, cyclopentyl‐, and cyclohexylfentanyl, as well as TMCPF, fentanyl, morphine, and buprenorphine, see Figure 1. All the potency and efficacy data can be found in Table 1.

**TABLE 1:** Efficacy and potency of alicyclic fentanyls. Adjusted *P* values are given for the difference in efficacy compared with fentanyl and values < 0.05 were considered significant. For potency, all differences are significant except for between cyclopropyl‐ and cyclobutylfentanyl. TMCPF, 2,2,3,3‐tetramethylcyclopropylfentanyl

	Efficacy			Potency		
	% of ref	95% CI (profile likelihood)	Adj. *P* values	LogEC_50_	EC_50_ nM	95% CI (profile likelihood)
Fentanyl	97	94–100	Ref	−8.76	1.7	1.4–2.2
Morphine	103	98–108	Ctrl	−6.37	430	330–550
Buprenorphine	74	71–78	Ctrl	−6.49	320	250–410
Cyclopropylfentanyl	97	91–103	Ns.	−8.37	4.3	2.5–7.1
Cyclobutylfentanyl	91	87–94	Ns.	−8.20	6.2	4.7–8.2
Cyclopentylfentanyl	92	89–95	Ns.	−7.88	13	10–17
TMCPF	65	61–68	< 0.0001	−5.82	1500	1200–1900
Cyclohexylfentanyl	48	45–51	< 0.0001	−5.50	3100	2400–4100

Cyclopropyl‐ (EC_50_ = 4.3 nM) and cyclobutylfentanyl (EC_50_ = 6.2 nM) were the most potent of the alicyclic fentanyls, being slightly less potent (*P* = 0.002 and <0.0001, respectively) than fentanyl (EC_50_ = 2.0 nM). Cyclopropyl‐ and cyclobutylfentanyl showed no significant difference in potency but significant differences in potency were observed for all other fentanyl analogs (adjusted *P* value < 0.05), in the following order (most potent first): fentanyl > cyclopropylfentanyl = cyclobutylfentanyl > cyclopentylfentanyl > TMCPF > cyclohexylfentanyl. Interestingly, both cyclohexylfentanyl and TMCPF were partial agonists, showing only 48% and 65% activation compared with fentanyl, respectively, see Table 1. Although not significant, trends for a lower efficacy were observed for cyclobutyl‐ (*P* value 0.07) and cyclopentylfentanyl (*P* value 0.16) compared with fentanyl.

## **4 |** DISCUSSION

In the present study, all alicyclic fentanyls tested showed activity at the μ‐opioid receptor indicating the potential for both abuse and fatal intoxication caused by respiratory depression.

As discussed by others, the measured K_i_ values from receptor binding studies are highly dependent on the assay conditions,^6^ and most likely this is also true for the EC_50_ values obtained in this study. To increase the relevance, the efficacies and potencies of the alicyclic fentanyls were compared with those of the well‐characterized agonists fentanyl, morphine, and buprenorphine.

To the best of our knowledge fentanyl, morphine, and buprenorphine have never been compared using an assay based on the aequorin system, but a few studies using human receptors have been conducted using other reporting systems. Lipinski et al^5^ and Hassanien et al^23^ reported binding affinities of fentanyl of 1.2 and 1.6 nM, respectively. Using a GTPγS binding assay, Olson et al^9^ used the same cell line as in this study and determined the EC_50_ values for morphine and buprenorphine to be 130 and < 0.1 nM, respectively with efficacies of 96% and 35% (compared with endomorphine‐2). Also using a GTPγS binding assay, Hassanien et al^23^ reported an EC_50_ value of 32 nM for fentanyl, while the Drug Enforcement Administration reported EC_50_ values of 32 nM and 17 nM for fentanyl and morphine, respectively. Using assays based on cAMP, Kuo et al^11^ reported IC_50_ values of 180, 0.2, and 1 nM for morphine, buprenorphine, and fentanyl, respectively, while Yu et al^10^ reported EC_50_ values of 5.3 and 0.02 nM for morphine and buprenorphine, respectively. Finally, Vasudevan et al reported an EC_50_ of 68 nM for fentanyl with an efficacy of 261% (compared with hydromorphone) using an assay based on the recruitment of mini‐G_i_.

As expected, the measured effective concentrations differed more than tenfold between assays, both between different types of assays but also between assays of the same type. For fentanyl, the EC_50_ value obtained in this study, 1.7 nM, is similar to data obtained from receptor binding studies (1.2 and 1.6 nM) as well as from the cAMP assay (1 nM), while lower than those obtained from GTPγS binding and mini‐Gi recruitment (32, 32, and 68 nM). Morphine on the other hand had an EC_50_ value of 430 nM compared with literature values in the range of 5–180 nM. Only two studies evaluated both fentanyl and morphine in the same model system. Yu et al^10^ reported fentanyl to be 180× more potent than morphine, while the Drug Enforcement Agency reported morphine to be more potent than fentanyl in their assay. This can be compared with the present study where fentanyl was 250× more potent than morphine. Three studies^9^, ^10^, ^11^ have evaluated both morphine and buprenorphine and all three studies reported buprenorphine to be around >250× more potent than morphine, while we found them to be equipotent. For reference, buprenorphine has previously been reported as 25–100 times more potent than morphine in vivo.^27^ The difference is surprising and the reason for this discrepancy is unknown. It might be related to the slow receptor dissociation reported for buprenorphine.^27^ In a flash assay, such as the one used in this study, with read times of a few seconds it is possible that the slow dissociation does not impact the assay in the same way as in cAMP and GTPγS binding assays with incubation times of an hour or more.

The five alicyclic fentanyls can be divided into two distinct groups. The three smaller analogs, cyclopropyl‐, cyclobutyl‐, and cyclopentylfentanyl were all full agonists with potencies similar to fentanyl, while cyclohexylfentanyl and TMCPF behaved like partial agonists of similar efficacy to buprenorphine but with lower potency. It was remarkable how suddenly the agonist behavior changed when going from a cyclopentyl ring to a cyclohexyl ring, and it is postulated that this might be due to steric hindrance at the binding site.

Except for cyclohexylfentanyl, the alicyclic fentanyl analogs have been studied before, although not together.^5^, ^12^, ^23^, ^24^ As there is considerable variability between the different assays the results were compared as fold changes from the EC_50_ value and the efficacy of fentanyl. In our study, cyclopropylfentanyl was a full agonist 2.5‐fold less potent than fentanyl. In other studies the EC_50_ varied from 2.9‐fold more potent to 1.7‐fold less potent than fentanyl with efficacies between 84% and 107% compared with fentanyl.^5^, ^12^, ^23^, ^24^ Cyclobutylfentanyl was a full agonist 3.1‐fold less potent than fentanyl in this study, while in the study by Hassanien et al^23^ it was 5.0‐fold less potent with an efficacy of 69% compared with fentanyl. Similarly, in our study cyclopentylfentanyl was a full agonist 7.6‐fold less potent than fentanyl, while in other studies it was 2.8 to 19‐fold less potent with an efficacy of 46–80% compared with fentanyl.^12^, ^23^ Finally, TMCPF behaved as a partial agonist (65% efficacy) 880‐fold less potent than fentanyl in our study and Vasudevan et al^12^ were unable to obtain a signal with either the mini‐Gi or the β‐arrestin assay. In general our EC_50_‐values compared with fentanyl are similar to those reported in previous studies, even though cyclopropylfentanyl appears somewhat less potent than previously reported. That said, when looking at the efficacies, cyclobutyl‐ and cyclopentylfentanyl appear to be full agonists in our study while they have previously been reported as partial agonists.

Based on the results, cyclopropyl‐, cyclobutyl‐, and cyclopentylfentanyl would be expected to induce intoxication or cause fatal poisonings at similar concentrations to fentanyl, while the concentrations of cyclohexylfentanyl and TMCPF required to cause intoxication would be expected to be much higher. That said, it is difficult to extrapolate from in vitro receptor activation to potency in vivo as the latter is also affected by important pharmacokinetic factors such as uptake, distribution, plasma binding, blood–brain barrier penetration, metabolism, and elimination. Even though analogs with lower potency and efficacy can still be abused, as illustrated by the high number of fatalities observed with buprenorphine,^28^ it is not possible to speculate on how high the concentrations of cyclohexylfentanyl and TMCPF are required to cause lethal respiratory failure and if such doses are forensically relevant. Interestingly, to the best of our knowledge, no deaths have been reported in the scientific literature attributed to these two analogs.

Earlier studies from our group have shown that the main routes of metabolism for alicyclic fentanyls include dealkylation to form normetabolites as well as oxidation of the alicyclic rings. With increasing ring size fewer normetabolites are formed, in favor of oxidation.^15^ If the oxidized metabolites maintain their activity while the normetabolites, analogous to norfentanyl, are inactive this could contribute to the observed potency and/or duration of effects of fentanyls with larger cyclic structures in vivo, but further experiments are needed to verify that this is the case.

## **5 |** CONCLUSIONS

In our study, using the AequoScreen assay, all alicyclic fentanyl analogs exhibited activity at the μ‐opioid receptor. Cyclopropyl‐, cyclobutyl‐, and cyclopentylfentanyl were all full agonists with a similar potency to fentanyl. On the contrary cyclohexylfentanyl and TMCPF were partial analogs of similar efficacy to buprenorphine but with lower potency.

## FUNDING INFORMATION

Vinnova, Eurostars Project ID: 113377 (the NPS‐reform project), and Strategic Research Area in Forensic Sciences (Strategiområdet forensiska vetenskaper) at Linköping University, grant numbers: 2016:4 and 2018:1.
